# Health trajectories across the work exit transition in the 1990s, 2000s, and 2010s: the role of working conditions and policy

**DOI:** 10.1186/s13690-022-01008-9

**Published:** 2023-02-06

**Authors:** Maaike van der Noordt, Theo G. van Tilburg, Suzan van der Pas, Bram Wouterse, Dorly J. H. Deeg

**Affiliations:** 1 Department of Epidemiology and Data Science, Amsterdam Public Health Research Institute, Amsterdam UMC, Vrije Universiteit Amsterdam, Amsterdam, the Netherlands; 2 Department of Health Knowledge Integration, Center for Health and Society, National Institute for Public Health and the Environment (RIVM), Bilthoven, The Netherlands; 3 Department of Sociology, Faculty of Social Sciences, Vrije Universiteit, Amsterdam, the Netherlands; 4 Faculty of Social Work and Applied Psychology, University of Applied Sciences Leiden, Leiden, Netherlands; 5 Erasmus School of Health Policy & Management, Erasmus University Rotterdam, Rotterdam, Netherlands

**Keywords:** Older workers, Self-rated health, Physical limitations, Workforce exit, Working conditions, Policy measures, Cross-sequential design

## Abstract

**Purpose:**

We examined health trajectories of Dutch older workers across their exit from the workforce in the 1990s, 2000s, and 2010s, testing the hypothesis that pre-post-exit health trajectories of workers with favourable and unfavourable working conditions increasingly diverged over time due to policy measures to extend working life.

**Methods:**

The Longitudinal Aging Study Amsterdam includes baseline samples in 1992/1993, 2002/2003 and 2012/2013 with two 3-year follow-up waves each. Selected respondents were aged 55 years and over who exited from a paid job within the first or second 3-year interval, up to and including the statutory retirement age (*N* = 522). Pre-post-exit trajectories were modelled using Generalized Estimating Equations with outcomes self-rated health and physical limitations and determinants physical demands, psychosocial demands, and psychosocial resources.

**Results:**

Average work exit age rose from 60.7 in the 1990s to 62.9 in the 2010s. On average, self-rated health decreased somewhat over successive periods and did not show pre-post-exit change; average physical limitations increased substantially both over successive periods and from pre- to post-exit. No support is found for our hypothesis. However, regardless of work exposures, we found sharp pre-post-exit increases in physical limitations in the 2010s.

**Conclusion:**

Although these findings provide no support for our hypothesis of diverging health trajectories over time based on work exposure, they show that exiting at a higher age is linked to poorer pre- and post-exit health and to pre-post-exit increases in physical limitations, suggesting greater health care costs in the near future.

**Supplementary Information:**

The online version contains supplementary material available at 10.1186/s13690-022-01008-9.

## Introduction

In many countries, policy reforms are aimed at delaying workforce exit of older workers in order to adjust to population ageing [1]. These reforms generally apply to all older workers, irrespective of their working conditions, i.e., their physical and psychosocial demands, and their psychosocial resources. With increasing age, workers need more time to recover from physically or psychosocially demanding tasks [2, 3]. Compared to younger workers, older workers are also more at risk of developing health problems from exposure to unfavourable working conditions [4]. Research in older workers shows that exposure to high physical or psychosocial work demands is associated with impaired health, as is exposure to working conditions that allow little autonomy [5, 6]. The question addressed in this study is whether the health consequences of prolonged exposure to unfavourable working conditions persist after exit from work. If this is the case, not only the quality of life of former workers will be affected, but poorer post-exit health is also likely to raise health care costs and thus offset the potential decrease in pension costs.

Health trajectories across the work exit transition of workers with different working conditions have not been studied extensively so far. Work exit itself does not have an unequivocal effect on health [7]. This unclarity may be due to the lack of consideration of work-related factors other than work exit itself, such as working conditions. Evidence on how the health effect of work exit depends on working conditions is not unequivocal either. French workers exposed to high physical and psychosocial work demands were in poorer pre-exit self-rated health, but their health increased more after the work exit transition compared to low exposure groups [8]. A Dutch study showed similar results regarding psychosocial demands [9]. Finnish workers with high exposure to physical demands had more physical limitations before and after retirement compared to those with lower exposure. However, these differences narrowed across the retirement transition as workers with high physical demands improved compared to lower exposure groups [10]. An early study suggested that workers with high physical demands may have benefited from the relief from these demands [11]. This suggestion was confirmed by more recent studies regarding mental, but not physical health [12, 13]. A Finnish study even showed that on average, self-rated health of workers remained stable across the retirement transition, but that individuals with high physical demands and job strain were at risk of health decline during this transition [14].

The past decades are characterised by the implementation of a series of policy measures aimed at extending working lives in many countries. In the Netherlands, measures have resulted in an increase in the average age of exit from the workforce [15]. For workers in the 1990s, early retirement was common and financially supported by employers and the Dutch government by means of early-retirement schemes. Early exit by older workers was intended to stimulate employment of younger workers and of newcomers to the labour market [16]. Older workers who were not covered by early-retirement schemes, were likely to use unemployment and disability insurance benefits as alternative pathways out of the labour market [17]. Since the early 2000s, social security programs and pension schemes have been reformed to extend the working lives of older adults. In the 2000s, early-retirement schemes were abolished, which made an early exit from the workforce financially unattractive. In addition, the government took measures to limit early exit by the alternative paths of unemployment and disability [18, 19]. However, the actual retirement age of workers in this period increased only marginally, most likely because numerous exceptions to the new measures were still in effect. In the 2010s, access to occupational disability schemes became more strictly limited and the maximum unemployment benefit period was limited [15]. A measure that affected all workers was the increase in the statutory retirement age, with its accompanying eligibility for a basic state pension. This increase occurred in steps from age 65 and one month in 2013 to age 66 and four months in 2019 [20].

It is an open question whether differences in pre-post-exit health trajectories remain the same over a period in which the actual retirement age has risen. A systematic review of the scarce literature on the health effects of the increase in the retirement age reports inconclusive evidence [21]. In particular, the question is whether the health of workers with favourable and unfavourable working conditions is affected differently. In the cited studies showing a more beneficial effect of retirement on health among workers with unfavourable working conditions compared to favourable working conditions, the average retirement age was low: between 55 and 60.5 years [8–10]. It is likely that workers who are exposed to unfavourable working conditions over a longer period of time and at higher ages, develop more health problems. Moreover, it might be too late for these workers to recover after retirement. For example, French retirees exposed to unfavourable working conditions aged 55 and over benefitted less from retirement than retirees aged younger than 55 years [8]. Finnish workers exposed to unfavourable working conditions were more at risk of health decline during the retirement transition compared to workers with favourable working conditions, and their average retirement age was over 62 years, i.e., they worked until older ages than workers in the studies showing a beneficial effect of retirement [14].

This study examines changes across three decades in health trajectories from pre- to post-exit in three cohorts of workers. We distinguish workers exposed to higher versus lower physical demands, psychosocial demands, and psychosocial resources. Because different health measures may yield different outcomes [22], we examine two health indicators: self-rated health and physical limitations. Our hypothesis is that, due to the increase in actual age of exit from the workforce, health trajectories across the work exit transition between workers exposed to higher versus lower levels of work demands and resources have started to diverge over the past decades. Specifically, we expect that workers exposed to higher physical and psychosocial demands and lower psychosocial resources have increasingly more unfavourable health trajectories than their counterparts with lower demands and higher resources. Insights from this study may contribute to the debate on the feasibility of further extension of working lives of workers in jobs with unfavourable working conditions [23], and adds the dimension of post-retirement health to this debate.

## Methods

### Study sample

The study sample is derived from the Longitudinal Aging Study Amsterdam (LASA), an ongoing Dutch cohort study investigating physical, emotional, cognitive and social functioning in late life [24, 25]. The first LASA cohort consists of 3,107 adults aged 55–85 in 1992/1993 who were interviewed face-to-face every three years, 964 of which were 55–64 years old (denoted as cohort 1). In 2002/2003 and 2012/2013, new cohorts were included with 996 (cohort 2) and 991 (cohort 3) adults aged 55–64, respectively, all with the same follow-up schedule. Data were gathered in face-to-face interviews in the participants' homes.

For this study, data from the first three waves (W1, W2, and W3) of each cohort were used. Exit from work was determined in two successive 3-year intervals, W1-W2 and W2-W3. In the combined cohorts, 1309 respondents did paid work (≥ 1 h/week) at W1, and 730 respondents at W2. Among the working respondents, drop-out due to mortality, refusal, intraceability or unknown work status at follow-up amounted to 253 (19.3%) between W1 and W2, and 82 (11.2%) between W2 and W3. From the remaining respondents, we included those who exited by W2 or W3 and whose age at exit was not higher than their statutory retirement age. A total of 601 respondents exited from work, for 596 of whom the age at exit was known. Applying the condition of exit before or at the statutory retirement age yielded a study sample of 522 respondents.

### Outcome

#### Health

Self-rated health was measured using a single self-report question: “How is your health in general?”, with five response categories ranging from very good to poor (scale 1–5) [26]. Physical limitations were measured using self-reports to six questions about difficulty or needing help doing six activities (scale 0–6). These activities concern climbing/descending stairs of 15 steps, getting dressed/undressed, sitting down on/standing up from a chair, cutting one’s toenails, walking outside for five minutes, and using public transportation [27]. The variable was dichotomised because of a right-skewed distribution, with the cut-off at ≥ 2.

### Determinants

#### Working conditions

Working conditions were assessed using the validated General Population Job-Exposure Matrix (GPJEM, [28]), which determines the level of work demands and resources of workers aged 55 and over based on the job description and its corresponding code of the Netherlands Standard Classification of Occupations 1992 [29]. Physical demands were based on the exposure probability of using a lot of force, working in an uncomfortable position, and making repetitive movements. Psychosocial demands were based on the exposure probability of working under much time pressure, having many task requirements, and experiencing cognitive demands, i.e., intensive thinking, need to keep focused, and requiring much concentration. Psychosocial resources were based on the exposure probability of having autonomy and variation in job activities. Physical demands and psychosocial resources scores ranged from 0 to 4, psychosocial demands ranged from 0 to 6. Higher scores indicate a higher exposure probability.

#### Work exit

Respondents were asked whether they had a paid job and if applicable, when (month and year) they exited from the work force. Thus, our definition of work exit covers all reasons of exit from paid work, i.e., 1) completely retired, 2) reached the statutory retirement age, 3) partially retired but no paid job, 4) unemployed (i.e., seeking a job), 5) on disability benefit, 6) inactive. Age at exit was calculated based on the month and year of exit from work and the birthdate obtained from the municipal registries. Time since exit was calculated by subtracting age at exit from age at follow-up.

#### Change over time

We distinguish two time variables: historic period and within-person time. The historic periods over which the three cohorts are followed, i.e., 1992/1993–1998/1999, 2002/2003–2008/2009, and 2012/2013–2018/2019, are denoted by the 1990s, 2000s, and 2010s, respectively. The corresponding period variable has values 0, 1, and 2. Within each cohort, the variable ‘within-person time’ denotes the time from pre-exit (value 0) to post-exit (value 1).

### Covariates

In addition to the work exit characteristics age at exit, time since exit, and reason for exit, other covariates were considered that may confound the association between historic period, working conditions, and pre-post-exit health change. Information on sex was obtained from the municipal registries. Educational level was self-reported and categorised into low (elementary education at most), middle (lower vocational and general intermediate education, intermediate vocational education and general secondary education) and high (higher vocational education, college education and university). The number of pre-exit working hours was self-reported and was included as a continuous variable. Age at exit had a non-linear association with self-rated health and therefore was categorised as 0) 55–58 years, 1) 59–63 years, and 2) 64 years through statutory retirement age.

### Analyses

Analyses were performed in SPSS 28. Statistical significance was set at *p* < 0.05; for interaction terms, at *p* < 0.10 [30].

First, we examined the characteristics of the study sample by period. To test whether pre- and post-exit characteristics changed over the periods, unweighted trend analyses using Chi-square tests were performed for categorical variables and one-way ANOVA tests for continuous variables.

Pre-post-exit trajectories were modelled using multivariate Generalized Estimating Equations (GEEs) with outcomes self-rated health and physical limitations. An exchangeable correlation structure was used to account for interdependency of repeated measurements within participants [31]. Analyses were performed for the two health indicators and the three working conditions separately. Self-rated health was examined with a linear model and physical limitations with a logistic model. Because the three work exposures proved to be strongly intercorrelated as well as correlated with educational level (*r* from 0.50 to 0.65), we used the residualised work exposures that resulted from linear regression of each working condition on educational level and the two other work exposures. This way, common variance among work exposures was excluded from the effect of a work exposure on both average health and change in health over time. The residualised working conditions were dichotomised at the mean. Each model consisted of one work exposure, within-person time, historic period, three two-way interaction terms, i.e., work exposure and within-person time, work exposure and period, and within-person time and period, and the three-way interaction term of work exposure, within-person time, and period, as well as the covariates sex, educational level, number of working hours, and age at exit. As the reason of exit in the successive periods may reflect policy measures, we did not include it in the models. We did not adjust for time since exit, because in preliminary analyses it did not affect the results.

Because of the presence of the interaction term of within-person time with work exposure, in each model for each period, the coefficient for the main effect of within-person time represents the health change for low demands or resources. For high demands or resources, health change is obtained by adding the coefficient of the interaction term of work exposure with within-person time to the coefficient of within-person time in the linear regression models, or by multiplying the odds ratios of within-person time with the odds ratio of the interaction term of work exposure with within-person time in the logistic regression models. The coefficient of the main effect of work exposure represents the effect of work exposure on pre-exit health. By changing the reference category of the period variable, we estimated a model for the work exposure * within-person time interaction for each period, with the covariates having equal effects [32].

Our hypothesis that health trajectories across the work exit transition between workers exposed to higher versus lower levels of work demands and resources have started to diverge over the past decades, to the disadvantage of workers exposed to higher physical and psychosocial demands and lower psychosocial resources, is then tested by examining whether the regression coefficients of the work exposure * within-person time interaction in the second or third period exceeded the corresponding 95% confidence intervals in an earlier period.

The health trajectories across the work exit transition by work exposure are also presented visually in graphs as estimated marginal mean scores (self-rated health) and probabilities (physical limitations).

## Results

### Descriptive findings

Table 1 shows pre- and post-exit characteristics of our study sample. The average statutory retirement age that applied for our study participants was 0.9 years higher in the 2010s than in earlier decades. Just over two-thirds of our study sample exited from work in the first time-interval, and less than one-third in the second interval – regardless of decade. The proportion of women was higher in the 2010s than earlier. The level of education rose gradually across the periods.

**Table 1:** Pre- and post-exit characteristics of workers who at exit age were younger than the statutory retirement age

	1990s	2000s	2010s	Total	*p*-value (linear)
N	150	176	196	522	
***General characteristics***					
Statutory retirement age (M, sd)	65.0	65.0	65.9 (0.5)	65.4 (0.6)	< 0.001
Exit in first 3-year interval (%)	67.3	64.8	74.0	69.0	0.152
Sex (% women)	40.7	42.6	54.6	46.6	0.008
Level of education (%)					
- Low	46.0	39.2	23.0	35.1	< 0.001
- Middle	32.7	34.7	43.9	37.5	
- High	21.3	26.1	33.2	27.4	
***Pre-exit work characteristics***					
Number or working hours/week (M, sd)^a^	32.2 (17.2)	27.5 (14.3)	27.9 (13.2)	29.0 (14.9)	0.007
Physical demands, range 0–4 (M, sd)	2.2 (1.5)	1.8 (1.6)	1.5 (1.6)	1.8 (1.6)	< 0.001
Psychosocial demands, range 0–6 (M, sd)	1.1 (1.9)	1.5 (1.9)	1.8 (2.1)	1.5 (2.0)	0.006
Psychosocial resources, range 0–4 (M, sd)	1.1 (1.3)	1.2 (1.3)	1.6 (1.4)	1.4 (1.4)	0.001
Residual physical demands (M, sd)	0.15 (1.09)	-0.06 (1.22)	-0.06 (1.19)	0.00 (1.17)	0.114
Residual psychosocial demands (M, sd)	-0.06 (1.43)	0.09 (1.30)	-0.04 (1.43)	0.00 (1.39)	0.955
Residual psychosocial resources (M, sd)	0.03 (0.89)	-0.09 (0.94)	0.06 (0.94)	0.00 (0.93)	0.657
***Pre-exit health***					
Self-rated health (M, sd)	2.0 (0.7)	2.2 (0.8)	2.2 (0.9)	2.1 (0.8)	0.200
Physical limitations (% > = 2)^b^	3.4	10.2	10.2	8.3	0.030
***Exit characteristics***					
Work exit age, continuous (M, sd)	60.7 (2.3)	61.3 (2.2)	62.9 (2.4)	61.7 (2.5)	< 0.001
Work exit age, categorical (%)					
- < = 58 years	22.7	13.6	9.2	14.6	< 0.001
- 59–63 years	64.7	72.7	49.0	61.5	
- > = 64 years	12.7	13.6	41.8	23.9	
Exit route^c^					
- Completely retired	50.7	51.7	36.9	46.2	< 0.001 (chi^2^)
- Reached statutory retirement age	14.7	17.0	21.6	17.9	
- Partly retired	4.7	1.1	4.0	3.2	
- Unemployed	4.0	6.8	14.8	8.8	
- Disability pension	10.7	13.6	5.7	10.0	
- Inactive	15.3	9.7	17.0	13.9	
Time after work exit in years (M, sd)	1.7 (0.8)	1.5 (0.9)	1.4 (0.9)	1.5 (0.9)	0.089
***Post-exit health***					
Self-rated health (M, sd)	2.1 (0.7)	2.2 (0.8)	2.2 (0.9)	2.1 (0.8)	0.651
Physical limitations (% > = 2)^d^	9.4	11.4	19.1	13.7	0.008

^a^ 1 case missing from cohort 1

^b^ 2 cases missing from cohort 1

^c^ 20 cases missing from cohort 3

^d^ 1 case missing from cohort 1, 2 cases missing from cohort 3

Regarding pre-exit work characteristics, the average number of working hours decreased after the 1990s. The average exposure to high physical demands decreased gradually, and exposure to both psychosocial demands and psychosocial resources increased gradually across the study period. Net of educational level and the other two work exposures, the work exposures showed no significant change. Thus, the rise in educational level can be considered to be largely responsible for the changes in average work exposures across the periods. The correlations between the residualised work exposures were small to moderate: physical demands correlated 0.03 and 0.45 with psychosocial demands and psychosocial resources, respectively, and psychosocial demands and resources correlated -0.41.

The average age at work exit increased from 60.7 years in the 1990s to 62.9 years in the 2010s. The type of exit was complete retirement in the majority of cases, with an increasing proportion retiring because of reaching the statutory retirement age rather than using early retirement schemes. Only a small proportion of workers was partially retired. The proportion of exits due to unemployment rose gradually and exits with disability pension dropped substantially in the 2010s.

Both pre-exit and post-exit self-rated health showed a non-significant increase across the decades. The proportion of workers with at least two physical limitations increased, both pre-exit and post-exit. Post-exit, physical limitations were higher than pre-exit. The correlation between the two health variables was moderate: 0.31 pre-exit and 0.39 post-exit.

### Working conditions

Below, we report the findings from our longitudinal models focusing on exposure to working conditions. Across work exposures, female gender, higher level of education, and longer working hours were associated with good self-rated health. Age younger than 59 at work exit was associated with poor self-rated health. Although in the same direction, these covariates did not always reach statistical significance regarding physical limitations. In these adjusted models, the association of period with health was practically always significant (p-value ranging from 0.012 to 0.076), indicating an increase in average poor health across the decades.

#### Physical demands

**Fig. 1:**
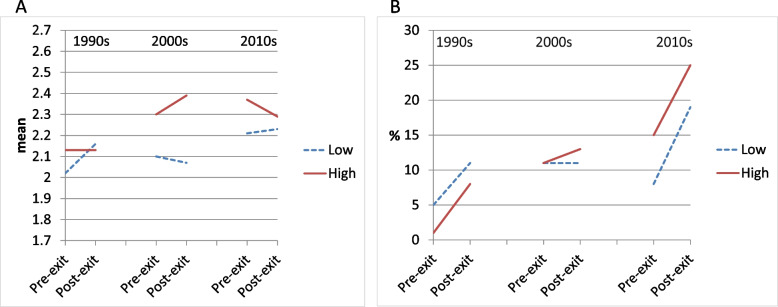
Changes in health pre-post-exit from the workforce by residualised ***physical demands*** during three periods: marginal estimates from models with covariates sex, educational level, baseline working hours (mean: 29.0). Solid lines: unfavourable exposures; dashed lines: favourable exposures. Panel A: Self-rated health (continuous, five categories from ‘very good’ to ‘poor’). Panel B: Physical limitations (difficulty or need of help with two or more activities)

Change in self-rated health across the exit from the workforce for workers with low physical demands was positive in the 1990s, indicating an overall decrease in self-rated health, although non-significant (Table 2, first row: B = 0.13, and Fig. 1a). In the 2000s and 2010s, however, change was virtually absent. Physical demands were associated with poorer pre-exit self-rated health, although the coefficient reached marginal significance only in the 2000s (Table 2, third row: B = 0.19). The interaction term of physical demands and within-person time was not statistically significant in any decade, indicating no differential pre-post-exit change in self-rated health for workers exposed to low and high physical demands (Table 2, third row). However, the coefficient of the 2000s interaction term (B = 0.13) was positive and significantly different from the coefficients of both the 1990s and 2010s interaction terms (B = -0.13 and -0.10, respectively), indicating divergence of pre-post health trajectories in the 2000s and convergence in the other decades. To illustrate, for workers exposed to low physical demands, the pre-post-exit change in self-rated health corresponded to B = -0.03 in the 2000s, whereas for workers exposed to high physical demands, this change was -0.03 + 0.13 = 0.10. In the 2010s, the health changes for these groups of workers were reversed with B = 0.02 and 0.02–0.10 = -0.08, respectively (Fig. 1a). Because the interaction term of physical demands, within-person time, and period was not significant (*p* = 0.225), however, our hypothesis is not supported.

**Table 2:** The association of pre-post exit health change with pre-exit **physical demands** by cohort (*N* = 522). General Estimating Equations with main effects, 2-way and 3-way interactions of cohort, **physical demands**, and within-person time. (The interaction terms including cohort are not shown, but their significance can be derived by comparing the B or OR across cohorts. See also Figs. 1–3.)

	1990s		2000s		2010s	
***Self-rated health (continuous)***						
	B	95% CI	B	95% CI	B	95% CI
Main part of model						
Time (post- vs pre-exit)	0.13	-0.05; 0.31	-0.03	-0.17; 0.10	0.02	-0.14; 0.18
Physical demands (high vs low)	0.10	-0.12; 0.33	0.19	-0.03; 0.41†	0.16	-0.08; 0.41
Physical demands * Time	-0.13	-0.36; 0.09	0.13^a^	-0.10; 0.36	-0.10^b^	-0.32; 0.12
**Adjustment variables (coefficients identical for the three decades)**						
Sex (female vs male)			-0.17	-0.31; -0.03*		
Level of education						
- High vs low			-0.23	-0.38; -0.08*		
-Middle vs low			-0.07	-0.21; 0.06		
Pre-exit working hours			-0.01	-0.01; -0.00*		
Exit age						
- < = 58 vs 59–63 years			0.22	0.03; 0.41*		
- > = 64 vs 59–63 years			0.01	-0.14; 0.16		
***Physical limitations***						
	OR	95% CI	OR	95% CI	OR	95% CI
Main part of model						
Time (post- vs pre-exit)	2.17	1.00; 4.73†	1.00	0.54; 1.84	2.57^b^	1.36; 4.85*
Physical demands (high vs low)	0.22	0.02; 2.20	1.03	0.39; 2.73	1.96	0.74; 5.22
Physical demands * Time	3.06	0.40; 23.58	1.27	0.52; 3.08	0.74	0.30; 1.83
**Adjustment variables (coefficients identical for the three decades)**						
Sex (female vs male)			0.72	0.39; 1.32		
Level of education						
- High vs low			0.26	0.12; 0.57**		
-Middle vs low			0.80	0.47; 1.39		
Pre-exit working hours			0.98	0.96; 1.00†		
Exit age						
- < = 58 vs 59–63 years			1.78	0.92; 3.45†		
- > = 64 vs 59–63 years			1.31	0.73; 2.36		

^**^ p < 0.001; * p < 0.05; † p < 0.10; B: unstandardized regression coefficient; OR: Odds Ratio; CI: Confidence Interval

^a^ Estimate differs significantly from estimate in 1990s

^b^ Estimate differs significantly from estimate in 2000s

Physical limitations for workers exposed to low physical demands showed an overall within-person increase across the exit from the workforce in the 1990s and the 2010s, but not in the 2000s. In the 2010s, this increase was significantly greater than in the 2000s (Table 2, lower panel, first row and Fig. 1b). Physical demands were not significantly associated with pre-exit physical limitations in any period. The interaction terms of physical demands and within-person time were non-significant in all periods, indicating that any increase in physical limitations was similar in workers with low and high physical demands. To illustrate, in the 2010s workers with low physical demands had an odd ratio of 2.57, indicating an increase in the odds of physical limitations over time. At the same time, workers exposed to high physical demands had an increase in odds of 2.57*0.74 = 1.90. This does not support our hypothesis that workers exposed to high physical demands would experience a poorer health trajectory compared to workers exposed to low physical demands. The interaction term of physical demands, within-person time, and period was not significant (*p* = 0.409).

#### Psychosocial demands

**Fig. 2:**
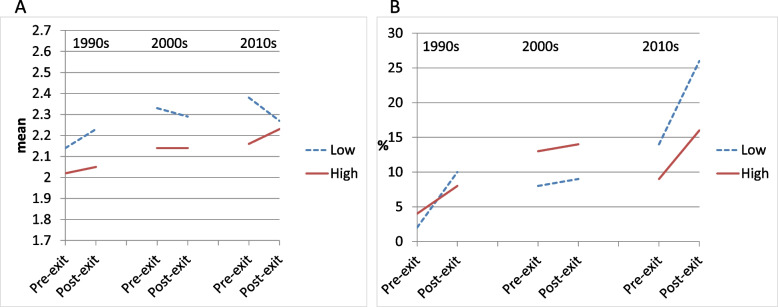
Changes in health pre-post-exit from the workforce by residualised ***psychosocial demands*** during three periods: marginal estimates from models with covariates sex, educational level, baseline working hours (mean: 29.0). Solid lines: unfavourable exposures; dashed lines: favourable exposures. Panel A: Self-rated health (continuous, five categories from ‘very good’ to ‘poor’). Panel B: Physical limitations (difficulty or need of help with two or more activities)

The regression coefficient for the effect of within-person time on self-rated health for workers with low psychosocial demands was positive in the 1990s and 2000s, and negative in the 2010s. The latter was significantly different from the earlier periods, indicating a reversal in the direction of the pre-post exit change from a decrease towards an increase in self-rated health for workers with low psychosocial demands in the 2010s (Table 3, first row and Fig. 2a). Psychosocial demands were negatively associated with poorer pre-exit self-rated health in all periods, but reached marginal significance only in the 2010s (Table 3, second row: B = -0.22). The interaction term of psychosocial demands and within-person time did not reach significance in any period. Comparing the coefficients for the interaction terms across the periods, the 2010s coefficient was positive and significantly different from earlier periods, again indicating a reversal: the self-rated health trajectories of workers with low and high psychosocial demands in the 2010s converged rather than diverged. This contradicts our hypothesis. The interaction term of psychosocial demands, within-person time, and period was not significant (*p* = 0.229).

**Table 3:** The association of pre-post exit health change with pre-exit **psychosocial demands** by cohort (*N* = 522). General Estimating Equations with main effects, 2-way and 3-way interactions of cohort, **psychosocial demands**, and within-person time. (The interaction terms including cohort are not shown, but their significance can be derived by comparing the B or OR across cohorts. See also Figs. 1–3.)

	1990s		2000s		2010s	
***Self-rated health (continuous)***						
	B	95% CI	B	95% CI	B	95% CI
Main part of model						
Time (post- vs pre-exit)	0.09	-0.09; 0.28	0.06	-0.10; 0.22	-0.11^ab^	-0.26; 0.04
Psychosocial demands (high vs low)	-0.11	-0.33; 0.10	-0.09	-0.32; 0.13	-0.22	-0.46; 0.02†
Psychosocial demands * Time	-0.06	-0.29; 0.17	-0.06	-0.29; 0.17	0.18^ab^	-0.04; 0.40
**Adjustment variables (coefficients identical for the three decades)**						
Sex (female vs male)			-0.18	-0.31; -0.04*		
Level of education						
- High vs low			-0.25	-0.40; -0.10**		
- Middle vs low			-0.16	-0.30; -0.02*		
Pre-exit working hours			-0.01	-0.01; -0.00**		
Exit age						
- < = 58 vs 59–63 years			0.20	0.01; 0.40*		
- > = 64 vs 59–63 years			-0.01	-0.16; 0.15		
***Physical limitations***						
	OR	95% CI	OR	95% CI	OR	95% CI
Main part of model						
Time (post- vs pre-exit)	6.82	1.05; 44.07*	1.19	0.56; 2.49	2.24	1.27; 3.93*
Psychosocial demands (high vs low)	2.69	0.28; 26.39	1.75	0.59; 5.18	0.60	0.22; 1.64
Psychosocial demands * Time	0.31	0.04; 2.34	0.93	0.36; 2.36	0.94	0.36; 2.40
**Adjustment variables (coefficients identical for the three decades)**						
Sex (female vs male)			0.67	0.37; 1.23		
Level of education						
- High vs low			0.26	0.12; 0.57**		
- Middle vs low			0.73	0.40; 1.32		
Pre-exit working hours			0.98	0.96; 1.00†		
Exit age						
- < = 58 vs 59–63 years			1.72	0.90; 3.28†		
- > = 64 vs 59–63 years			1.30	0.72; 3.35		

^**^ p < 0.001; * p < 0.05; † p < 0.10; B: unstandardized regression coefficient; OR: Odds Ratio; CI: Confidence Interval

^a^ Estimate differs significantly from estimate in 1990s

^b^ Estimate differs significantly from estimate in 2000s

Physical limitations showed significant increases in the 1990s and 2010s for workers with low psychosocial demands (Table 3, lower panel, first row, and Fig. 2b). Psychosocial demands were not significantly associated with poorer pre-exit physical limitations in any period. All interaction terms were non-significant, showing that pre-post-exit trajectories in physical limitations were not significantly different for workers with low and high psychosocial demands. The interaction term of psychosocial demands, within-person time, and period was also not significant (*p* = 0.600). Thus, for psychosocial demands, our hypothesis was not supported.

#### Psychosocial resources

**Fig. 3:**
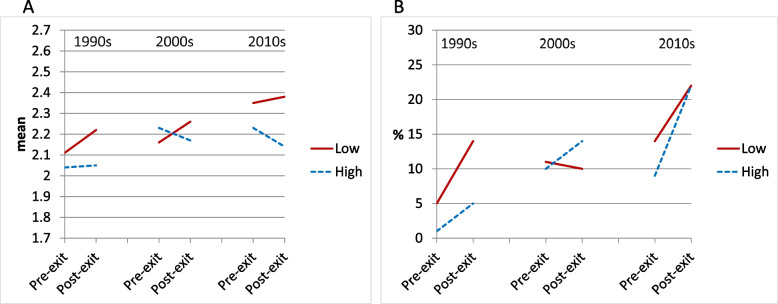
Changes in health pre-post-exit from the workforce by residualised ***psychosocial resources*** during three periods: marginal estimates from models with covariates sex, educational level, baseline working hours (mean: 29.0). Solid lines: unfavourable exposures; dashed lines: favourable exposures. Panel A: Self-rated health (continuous, five categories from ‘very good’ to ‘poor’). Panel B: Physical limitations (difficulty or need of help with two or more activities)

In the model for psychosocial resources, the pre-post-exit change in self-rated health for workers with low psychosocial resources was positive for all periods, indicating poorer self-rated health over time. However, the coefficients for within-person time were never significant, and also did not significantly differ across periods (Table 4, first row and Fig. 3a). Psychosocial resources did not show a significant association with pre-exit self-rated health in any period. The interaction terms of psychosocial resources and within-person time were negative, indicating a more favourable trajectory in self-rated health among workers with high exposure to psychosocial resources than among workers with low exposure to psychosocial resources. However, the interaction term did not reach significance in any period (Table 4, third row). Thus, these findings do not support our hypothesis of increasing divergence. The interaction term of psychosocial resources, within-person time and period was far from significant (*p* = 0.926).

**Table 4:** The association of pre-post exit health change with pre-exit **psychosocial resources** by cohort (*N* = 522). General Estimating Equations with main effects, 2-way and 3-way interactions of cohort, **psychosocial resources**, and within-person time. (The interaction terms including cohort are not shown, but their significance can be derived by comparing the B or OR across cohorts. See also Figs. 1–3.)

	1990s		2000s		2010s	
***Self-rated health (continuous)***						
	B	95% CI	B	95% CI	B	95% CI
Main part of model						
Time (post- vs pre-exit)	0.12	-0.07; 0.30	0.10	-0.04; 0.24	0.03	-0.14; 0.20
Psychosocial resources (high vs low)	-0.07	-0.28; 0.15	0.08	-0.14; 0.30	-0.12	-0.37; 0.12
Psychosocial resources * Time	-0.10	-0.33; 0.12	-0.17	-0.40; 0.07	-0.12	-0.34; 0.10
**Adjustment variables (coefficients identical for the three decades)**						
Sex (female vs male)			-0.21	-0.35; -0.06*		
Level of education						
- High vs low			-0.24	-0.39; -0.09*		
- Middle vs low			-0.11	-0.25; 0.02		
Pre-exit working hours			-0.01	-0.01; -0.00*		
Exit age						
- < = 58 vs 59–63 years			0.22	0.02; 0.41*		
- > = 64 vs 59–63 years			-0.00	-0.16; 0.15		
***Physical limitations***						
	OR	95% CI	OR	95% CI	OR	95% CI
Main part of model						
Time (post- vs pre-exit)	2.81	1.22; 6.59*	0.89^a^	0.44; 1.80	1.73	0.97; 3.08†
Psychosocial resources (high vs low)	0.21	0.02; 1.99	0.90	0.34; 2.40	0.64	0.24; 1.68
Psychosocial resources * Time	1.52	0.21; 10.91	1.64	0.67; 3.99	1.64	0.65; 4.14
**Adjustment variables (coefficients identical for the three decades)**						
Sex (female vs male)			0.65	0.35; 1.20		
Level of education						
- High vs low			0.27	0.12; 0.57**		
- Middle vs low			0.74	0.44; 1.24		
Pre-exit working hours			0.98	0.96; 1.00†		
Exit age						
- < = 58 vs 59–63 years			1.77	0.92; 3.41†		
- > = 64 vs 59–63 years			1.28	0.71; 2.30		

^**^ p < 0.001; * p < 0.05; † p < 0.10; B: unstandardized regression coefficient; OR: Odds Ratio; CI: Confidence Interval

^a^ Estimate differs significantly from estimate in 1990s

^b^ Estimate differs significantly from estimate in 2000s

Regarding physical limitations, a significant increase was observed for workers with low psychosocial resources in the 1990s and 2010s, but not in the 2000s (Table 4, lower panel and Fig. 3b). The odds ratios for psychosocial resources were smaller than 1 in all periods, suggesting a protective effect against pre-exit physical limitations. However, none of these reached significance. The interaction terms of psychosocial resources with within-person time were positive in all periods, but never reached significance. Thus, workers with low and high psychosocial resources did not show different pre-post-exit trajectories in physical limitations and again, our hypothesis was not supported. The interaction term of psychosocial resources, within-person time and period was far from significant (*p* = 0.997).

## Discussion

In this study, we examined health trajectories in self-rated health and physical limitations across the work exit transition for older workers exposed to higher versus lower physical demands, psychosocial demands, and psychosocial resources. We did this for the 1990s, 2000s, and 2010s, i.e., during a period in which Dutch policy measures to extend working lives accumulated and a steady increase in the age at exit from the workforce took place. We hypothesized that differences in health trajectories between workers exposed to less favourable and more favourable working conditions increased from the 1990s to the 2010s. Our hypothesis was not supported, neither for self-rated health nor for physical limitations. Regardless of work exposure, we did observe that for both health measures, the level of poor health was somewhat higher in later than in earlier decades. Changes in pre-post-exit health were not observed for self-rated health. In physical limitations, however, pre-post-exit increases were observed in the 1990s and 2010s regardless of work exposure, whereby the increase was especially steep in the 2010s.

Our hypothesis of gradually diverging health trajectories for workers with favourable and unfavourable work exposures was based on our expectation of a cumulative effect of policy measures that were implemented since the early 2000s. The lack of support for our hypothesis calls for a closer look at the successive policy measures and their effects on pre-post exit health trajectories. At our starting point in the 1990s, many workers used one of the several opportunities to leave the workforce well before the statutory retirement age, including early retirement schemes and disability pensions [16, 17]. This is reflected in our data by the low level of physical limitations in workers in the 1990s. In the 2000s, exiting from the workforce via disability pensions was restricted, but most important was the abolishment of early retirement schemes [18, 19]. Thus, both workers with favourable and workers with unfavourable working conditions extended their working lives. Our findings for the 2000s show that this did not coincide with a significant divergence in pre-post-exit health trajectories. However, both groups of workers tended to report poorer self-rated health and a higher level of physical limitations than in the 1990s. In the 2010s, to the extant policy measures a gradual rise in the statutory retirement age was added, access to disability benefits was further restricted, and the maximum unemployment benefit period was limited [15, 20]. At the same time, the measures taken in the 2000s reached their full effect, so that it became increasingly necessary to continue working. We again observed a slightly poorer self-rated health in the 2010s. The latter finding may reflect that older workers had expected to exit earlier [33]. The frustration of having to work for more years may negatively affect their perception of their health, even after exit from the workforce. Regarding physical limitations, their pre-exit level was similar to the level in the previous decade, but their pre-post-exit increase was substantial and contrasted with the stability of physical limitations in the 2000s. Thus, in the 2010s workers did not recover from their pre-exit physical limitations, but became more limited after exiting from work. This is consistent with evidence that with increasing age, older workers become more vulnerable to any work exposure, regardless of level of education, and that recovery becomes more difficult even after elimination of work exposures [2–4].

Another possible reason that our hypothesis of diverging health trajectories does not hold, is that the effect of working conditions was counteracted by adjustments. Our descriptive data (Table 1) show that over the decades, the average number of working hours decreased. In our analytic models, shorter working hours were associated with poorer health. Reducing the number of working hours is one strategy to boost workability [34]. Furthermore, the correlation of physical demands with psychosocial resources was positive, while a negative association would have been expected: workers exposed to high physical demands are likely to have jobs with little autonomy and variation. A tentative explanation may be that our samples include older workers in physically demanding jobs who have been able to remain in the workforce thanks to the availability of a relatively high level of psychosocial resources. Regardless, even with adjustments in working hours and the possible presence of psychosocial resources, in the 2010s physical health declined.

In sum, it appears that working conditions did not play the hypothesised role, suggesting that their potential effects on pre-post-exit health trajectories were overshadowed by more powerful influences. Because new retirement policy measures were implemented in the course of the decades studied, it seems reasonable to attribute the observed health decline to these new policies, which effectively increased the age at exit from the labour market. In other words, it seems reasonable to attribute our findings to period effects rather than to cohort effects. Period effects affect all individuals living in a certain historic period, whereas cohort effects result from influences associated with cohort membership and may have their origin earlier in the life course [35]. In our analyses we attempted to rule out cohort effects by adjusting for some major cohort characteristics, including level of education, sex, and number of working hours. These characteristics capture the rise in educational level and in women’s – often part-time – labour market participation across cohorts. However, unobserved cohort or period effects may have influenced our findings as well. Before our findings are replicated in future research, caution is warranted in stressing policy measures as the only influence.

Several specific findings deserve discussion. Corresponding to the policy measures taken earlier, our data show that in the 2010s substantially fewer workers exited with a disability benefit scheme. We did not adjust our models for type of exit, because this would preclude the reflection of policy measures in our findings. Yet, this also precluded an assessment of the extent to which workers who exited with a disability scheme affected the health trajectories that we found. As expected, our data show that these workers had poorer health both pre- and post-exit compared to workers with other exit routes (Table S1). Therefore, we reanalysed our models excluding the workers with a disability exit (*n* = 50; Supplementary tables S2-S4). This did not essentially change our findings: although at slightly better levels of health, we still observed poorer levels of self-rated health across the successive periods, and a substantial increase in physical limitations in the 2010s regardless of working conditions. The similarity in findings when including and excluding disability exits ties in with the findings from a Swedish study, which showed that the relationship between exit route and post-exit health disappeared once pre-exit health was added to the model [36]. These researchers concluded that both exit route and post-exit health can be considered as outcomes of a lifelong process of accumulation of differential work exposures. This implies that the type of exit has no added value to pre-post exit health trajectories, provided that pre-exit health is properly accounted for.

According to the life course perspective, working conditions are a result of selection into certain occupations, which is amongst others based on prior levels of education [37]. It was our intention to examine working conditions per se, unconfounded by level of education. As in our study working conditions turned out to have little effect on health trajectories, it may be argued that the accumulation of earlier exposures culminate in pre-exit work exposures, but that these work exposures are not in themselves causal. In order to test if the role of educational level is predominant, we conducted additional analyses defining level of education as the work exposure instead of working conditions. The results were very similar to our original analyses, with the exception that physical ﻿limitations showed a steeper pre-post exit rise in lower than in higher educated workers in the 2010s (Table S5). This analysis stresses the close link between educational level and later-life working conditions.

In our study, workers with high psychosocial demands reported better self-rated health than workers with low psychosocial demands. This contrasts with studies showing that psychosocial demands negatively affect health. This may be due to the inclusion of different items in the psychosocial demands scale. For example, we included cognitive demands in addition to time pressure and work load, whereas other studies included only the latter two items (e.g., [9]). Workers experiencing high cognitive demands are likely to have higher-status jobs, and job status is positively associated with good self-rated health (e.g., [14]).

Pre-post-exit trajectories were different for self-rated health and for physical limitations. Particularly in the 2010s, the latter increased across the exit from the workforce, but the former remained stable. Possibly, a certain relief after exiting from work plays a role [12, 13], which may compensate for increases in physical limitations. Self-rated health is known to be influenced by both physical and mental health [38], and in contrast to mixed evidence on physical health, mental health has been shown to improve after work exit [7, 22]. Another potential explanation for the relatively flat trajectories of self-rated health as opposed to physical limitations is the phenomenon of response shift [39]. This phenomenon entails that in the face of health problems, people lower their standard of good health, so that their experience of their health remains stable.

### Strengths and limitations

A strength of this study is the use of a representative sample of the older working population. In addition, the cohort-sequential design of LASA enabled us to compare the health trajectories during different time periods. The distinction of different periods is novel and adds to the literature, where other studies aggregated findings over historic time (e.g., [40, 41]).

Limitations of our study include, first, the relatively low number of respondents eligible for our study sample. However, our sample was representative of the general older population. This is supported by a comparison of the average age at retirement in our sample with national data from Statistics Netherlands. At the national level, for persons aged 60–65 the average retirement age was 60.8 in the late 1990s, which compares well with our average age of 60.7 years for the 1990s, as the retirement age did not change in this decade. In the mid-2010s, the average national retirement age for this age group was 64.4 [42]. This is somewhat higher than our 62.9 years, but our sample was capped at the statutory retirement age, so that the exit age of those who worked beyond statutory retirement age did not raise the average. Regardless, our results need to be confirmed using larger samples.

Second, the health trajectories were based on two waves, one before and one after work exit. This allowed inclusion of a maximal number of participants in each decade. By using more than two waves, we could have addressed the possibility that health trajectories differ pre- and post-exit [40] or are more heterogeneous post-exit [41]. The availability of only one wave pre-exit precluded the possibility to determine if working conditions had changed prior to this wave. It is possible that the pre-exit working conditions no longer reflect the work exposures during the longest-held job. Likewise, the availability of only one post-exit wave also precluded the possibility to differentiate short-term from long-term health trajectories. Schmälzle and colleagues [41], for example, found that life satisfaction tended to decline pre-exit, but showed a certain recovery at one year post-exit. As in our study the time interval between waves was three years, inclusion of more waves per participant pre- and post-exit would have discarded the clear separation between decades, while the main objective of our study was to address decade-specific trajectories.

Third, working conditions are derived from a General Population Job-Exposure Matrix (GPJEM) for workers aged 55 and over [28]. This GPJEM does not take heterogeneity within job categories into account. However, the advantage of a GPJEM is that it is not influenced by individual characteristics, such as health, which may lead to reversed causality [43] or common-method bias [44]. Furthermore, our GPJEM is developed based on working conditions reported by workers over the period 2005–2010 [28]. Therefore, it is possible that it does not correctly capture the working conditions in the 1990s and 2010s. For example, the same job may have higher physical demands in the 1990s and lower physical demands in the 2010s due to technological developments [45, 46]. Also, to keep older workers in the workforce, the working conditions of workers showing declines in work ability may have been adapted while the job description remained the same. However, this is not very likely, as employers have been shown to prefer retiring older workers over offering work adaptations [47]. Although both self-reported and GPJEM-based working conditions have biases, arguably a GPJEM describes working conditions more objectively.

## Conclusion

Our study provides no support for our hypothesis of diverging health trajectories based on work exposure. What our study does show clearly is that exiting at a higher age, which was at least partly induced by policy measures in the 2010s, is linked to poorer pre- and post-exit health and to pre-post-exit increases in physical limitations. The deterioration of health accompanying exit from the workforce is critical for both individuals and society. Ex-workers in poor health are less likely to participate in society through voluntary work and informal care [48, 49]. In addition, poorer post-retirement health leads to greater health care expenditures [50, 51]. Post-exit health may be improved by improving the working conditions of workers at older ages, by an adapted statutory retirement age for workers with unfavourable working conditions, and by flexible retirement schemes [52, 53]. In view of the ongoing increase in the average retirement age in the Netherlands, our findings stress the importance of monitoring both pre- and post-exit health in workers [54].

## Supplementary Information

**Additional file 1: Table S1.** Pre- and post-exit characteristics of workers by exit route. **Table S2.** The association of pre-post exit health change with pre-exit physical demands by cohort, excluding disability exits (N=451†). **Table S3.** The association of pre-post exit health change with pre-exit psychosocial demands by cohort, excluding disability exits (N=451). **Table S4.** The association of pre-post exit health change with pre-exit psychosocial resources by cohort, excluding disability exits (N=451). **Table S5.** The association of pre-post exit health change with pre-exit educational level by cohort (N=522)

## Data Availability

The dataset analysed during this study are available under the rules that are valid for the Longitudinal Aging Study Amsterdam (www.lasa-vu.nl), i.e., data are available for use for specific research questions provided that an agreement is made up. Contact: lasa@amsterdamumc.nl.

## References

[1] European Commission. White paper: an agenda for adequate, safe and sustainable pensions. European Commission; 2012.

[2] Kiss P, De Meester M, Braeckman L (2008). Differences between younger and older workers in the need for recovery after work. Int Arch Occup Environ Health..

[3] Verdonk P, Hooftman WE, van Veldhoven MJPM, Boelens LRM, Koppes LLJ (2010). Work-related fatigue: the specific case of highly educated women in the Netherlands. Int Arch Occup Environ Health.

[4] Jones MK, Latreille PL, Sloane PJ, Staneva AV (2013). Work-related health risks in Europe: are older workers more vulnerable?. Soc Sci Med.

[5] Andrasfay T, Raymo N, Goldman N, Pebley AR (2021). Physical work conditions and disparities in later life functioning: potential pathways. SSM Pop Health.

[6] de Breij S, Huisman M, Deeg DJH (2020). Work characteristics and health in older workers: educational inequalities. PLoS ONE.

[7] van der Heide I, van Rijn R, Robroek SJW, Burdorf A, Proper K (2013). Is retirement good for your health? A systematic review of longitudinal studies. BMC Public Health.

[8] Westerlund H, Kivimäki M, Singh-Manoux A, Melchior M, Ferrie JE, Pentti J, et al. Self-rated health before and after retirement in France (GAZEL): A cohort study. Lancet. 2009;374:1889–96. 10.1016/S0140-6736(09)61570-1. 10.1016/S0140-6736(09)61570-1 19897238

[9] van den Bogaard L, Henkens K, Kalmijn M (2016). Retirement as a relief? The role of physical job demands and psychological job stress for effects of retirement on self-rated health. Eur Sociol Rev..

[10] Mänty M, Kouvonen A, Lallukka T, Lahti J, Lahelma E, Rahkonen O (2016). Pre-retirement physical working conditions and changes in physical health functioning during retirement transition process. Scand J Work Environ Health..

[11] Ekerdt DJ, Bosse R, LoCastro JS (1983). Claims that retirement improves health. J Gerontol..

[12] Jokela M, Ferrie JE, Gimeno D, Chandola T, Shipley MJ, Head J (2010). From midlife to early old age: health trajectories associated with retirement. Epidemiol (Cambridge MA.).

[13] Mein G, Martikainen P, Hemingway H, Stansfeld S, Marmot M (2003). Is retirement good or bad for mental and physical health functioning? Whitehall II longitudinal study of civil servants. J Epidemiol Community Health.

[14] Stenholm S, Virtanen M, Pentti J, Oksanen T, Kivimäki M, Vahtera J (2020). Trajectories of self-rated health before and after retirement: evidence from two cohort studies. Occup Environ Med..

[15] Atav T, Jongen E, Rabaté S. The effects of the increase in the retirement age in the Netherlands. CPB Discussion paper. Neth Bur Econ Pol Anal 2016.

[16] Council Socio-Economic (1999). Bevordering arbeidsdeelname ouderen [Promotion of labour participation of older people].

[17] Lindeboom M (1996). Vervroegde uittreding uit de arbeidsmarkt: een empirische analyse naar de determinanten van stoppen met werken [Early exit from the labour market: an empirical analysis of the determinants of stopping with work]. Tijdschr Politieke Econ.

[18] OECD (Organisation for Economic Co-operation and Development). Sickness and disability schemes in the Netherlands. Country memo as a background paper for the OECD Disability Review. Paris: OECD Publishing. 2007.

[19] van Oorschot W (2007). Narrowing pathways to early retirement in the Netherlands. Benefits.

[20] Government (2019). AOW-leeftijd op basis van principeakkoord juni 2019 [Statutory retirement age based on the principle agreement June 2019].

[21] Pilipiec P, Groot W, Pavlova M (2021). The effect of an increase of the retirement age on the health, well-being, and labor force participation of older workers: a systematic literature review. J Pop Ageing.

[22] van den Bogaard L, Henkens K (2018). When is quitting an escape? How different job demands affect physical and mental health outcomes of retirement. Eur J Public Health..

[23] Natali D, Spasova S, Vanhercke B (2016). Retirement regimes for workers in arduous or hazardous jobs in Europe.

[24] Hoogendijk EO, Deeg DJH, Poppelaars J, van der Horst M, Broese van Groenou MI, Comijs HC (2016). The Longitudinal Aging Study Amsterdam: cohort update 2016 and major findings. Eur J Epidemiol.

[25] Huisman M, Poppelaars J, van der Horst M, Beekman A, Brug J, van Tilburg T (2011). Cohort profile: The Longitudinal Aging Study Amsterdam. Int J Epidemiol.

[26] Jylhä M (2009). What is self-rated health and why does it predict mortality? Towards a unified conceptual model. Soc Sci Med.

[27] McWhinnie JR (1981). Disability assessment in population surveys: results of the OECD common development effort. Rev Epidemiol Sante Publique.

[28] Rijs KJ, van der Pas S, Geuskens GA, Cozijnsen R, Koppes LJJ, Van der Beek AJ (2014). Development and validation of a physical and psychosocial job-exposure matrix in older and retired workers. Ann Occup Hyg.

[29] Statistics Netherlands (2001). Standaard beroepenclassificatie 1992 [Netherlands standard classification of occupations 1992].

[30] Aiken L, West S, Reno R (1991). Multiple regression: testing and interpreting interactions.

[31] Twisk JWR (2013). Applied Longitudinal Data Analysis for Epidemiology: A Practical Guide.

[32] Figueiras A, Domenech-Massons JM, Cadarso C (1998). Regression models: calculating the confidence interval of effects in the presence of interactions. Stat Med..

[33] Henkens K, van Solinge H, Damman M, Dingemans E (2016). Taken by surprise: How older workers struggle with a higher retirement age. Demos..

[34] Varekamp I, van Dijk FJH (2010). Workplace problems and solutions for employees with chronic diseases. Occup Med.

[35] Glenn ND (1976). Cohort analysts’ futile quest: statistical attempts to separate age, period, and cohort effects. Amer Sociol Rev.

[36] Halleröd B, Örestig J, Stattin M (2013). Leaving the labour market: the impact of exit routes from employment to retirement on health and wellbeing in old age. Eur J Ageing.

[37] Ravesteijn B, van Kippersluis H, van Doorslaer E (2018). The wear and tear on health: What is the role of occupation?. Health Econ.

[38] Pinquart M (2001). Correlates of subjective health in older adults: a meta-analysis. Psychol Aging.

[39] Galenkamp H, Huisman M, Braam AW, Deeg DJ (2012). Estimates of prospective change in self-rated health in older people were biased owing to potential recalibration response shift. J Clin Epidemiol.

[40] Marshall A, Nazroo J (2016). Trajectories in the prevalence of self-reported illness around retirement. J Pop Ageing.

[41] Schmälzle M, Wetzel M, Huxhold O (2019). Pathways to retirement: are they related to patterns of short- and long-term subjective well-being?. Soc Sci Res..

[42] Statistics Netherlands. Van arbeid naar pensioen; personen 55 jaar of ouder, 1999-2016 [From labour to retirement; persons aged 55 years and older, 1999-2016]. 22 March 2017. Available from: http://statline.cbs.nl/Statweb/publication/?DM=SLNL&PA=80396ned&D1=1,3-5,7&D2=0&D3=0&D4=0&D5=1&D6=0&D7=a&D8=2-16&HDR=T,G6&STB=G1,G2,G3,G4,G5,G7&VW=T.

[43] Landsbergis P (2000). Measurement of psychosocial workplace exposure variables. Occup Med.

[44] Podsakoff PM, MacKenzie SB, Lee J-Y, Podsakoff NP (2003). Common method biases in behavioral research: a critical review of the literature and recommended remedies. J Appl Psychol.

[45] Näswall K, Hellgren J, Sverke M (2008). The individual in the changing working life.

[46] Romeu Gordo L, Skirbekk V (2013). Skill demand and the comparative advantage of age: jobs tasks and earnings from the 1980s to the 2000s in Germany. Labour Econ.

[47] van Dalen H, Henkens K, Wang M (2015). Recharging or retiring older workers? Uncovering the age-based strategies of European employers. Gerontologist.

[48] Sabbath EL, Lubben J, Goldberg M, Zins M, Berkman LF (2015). Social engagement across the retirement transition among “young-old” adults in the French GAZEL cohort. Eur J Ageing..

[49] Schmidt AE, Ilinca S, Schulmann K, Rodrigues R, Principi A, Barbadella F (2016). Fit for caring: factors associated with informal care provision by older caregivers with and without multimorbidity. Eur J Ageing.

[50] Johar M (2017). The evolution of out-of-hospital medical costs to and through retirement. Econ Papers..

[51] Wouterse B, Huisman M, Meijboom BR, Deeg DJ, Polder JJ (2013). Modeling the relationship between health and health care expenditures using a latent Markov model. J Health Econ..

[52] Boot CRL, van den Heuvel SG, Bültmann U, de Boer AGEM, Koppes LLJ, van der Beek AJ (2013). Work adjustments in a representative sample of employees with a chronic disease in the Netherlands. J Occup Rehabil.

[53] Vermeer N, Mastrogiacomo M, van Soest A (2016). Demanding occupations and the retirement age. Labour Econ..

[54] OECD (Organisation for Economic Co-operation and Development). Ageing and employment policies: Netherlands, (2014). Working better with age.

