# Supplementary material for: Health trajectories across the work exit transition in the 1990s, 2000s, and 2010s: the role of working conditions and policy
Source: Arch Public Health. 2023 Feb 6;81:16. doi: 10.1186/s13690-022-01008-9 (PMC9901107; doi:10.1186/s13690-022-01008-9)
Supplement: Supplementary file 1 — Additional file 1: Table S1. Pre- and post-exit characteristics of workers by exit route. Table S2. The association of pre-post exit health change with pre-exit physical demands by cohort, excluding disability exits (N=451†). Table S3. The association of pre-post exit health change with pre-exit psychosocial demands by cohort, excluding disability exits (N=451). Table S4. The association of pre-post exit health change with pre-exit psychosocial resources by cohort, excluding disability exits (N=451). Table S5. The association of pre-post exit health change with pre-exit educational level by cohort (N=522) [file 13690_2022_1008_MOESM1_ESM.docx]

**Health trajectories across the work exit transition in the 1990s, 2000s, and 2010s: the role of working conditions and policy**

Maaike van der Noordt, Theo G. van Tilburg, Suzan van der Pas, Bram Wouterse, Dorly J.H. Deeg

**Corresponding author:** Prof. dr. Dorly J.H. Deeg, Amsterdam UMC, Vrije Universiteit Amsterdam, Department of Epidemiology and Biostatistics, Amsterdam Public Health Research Institute, Amsterdam, the Netherlands. E-mail: djh.deeg@amsterdamumc.nl

**SUPPLEMENTARY MATERIAL**

**Table S1**. Pre- and post-exit characteristics of workers by exit route

|  | **Completely retired**  **(n=241)** | **Reached SRA**  **(n=93)** | **Partly retired**  **(n=16)** | **Unemployed**  **(n=45)** | **Disability pension**  **(n=50)** | **Unknown**  **(n=77)** | ***p*-value** |
| --- | --- | --- | --- | --- | --- | --- | --- |
| Sex (% female) | 33.2 | 52.7 | 43.8 | 48.9 | 32.0 | 76.6 | <0.001 |
| Education (% low) | 31.1 | 32.3 | 37.5 | 15.6 | 46.0 | 53.2 | 0.001 |
| No of working hrs (M, sd) | 32.9 (12.9) | 25.3 (16.3) | 32.1 (12.3) | 30.5 (12.0) | 26.5 (14.8) | 22.2 (17.5) | <0.001 |
| Exit age (M, sd) | 61.3 (2.1) | 64.6 (0.6) | 61.1 (2.6) | 60.3 (2.0) | 60.0 (2.1) | 61.3 (2.1) | <0.001 |
|  |  |  |  |  |  |  |  |
| Physical demands, range 0-4 (M, sd) | 1.7 (1.5) | 1.6 (1.6) | 1.4 (1.7) | 1.3 (1.5) | 2.3 (1.7) | 2.4 (1.5) | <0.001 |
| Psychosocial demands, range 0-6 (M, sd) | 1.8 (2.1) | 1.6 (2.1) | 1.7 (2.1) | 1.5 (1.9) | 1.4 (1.8) | 0.6 (1.4) | <0.001 |
| Psychosocial resources, range 0-4 (M, sd) | 1.6 (1.3) | 1.4 (1.5) | 1.5 (1.7) | 1.7 (1.4) | 1.0 (1.3) | 0.7 (1.0) | <0.001 |
| Residual physical demands (M, sd) | 0.07 (1.16) | -0.18 (1.17) | -0.37 (1.51) | -0.17 (1.14) | 0.28 (1.13) | -0.00 (1.02) | 0.148 |
| Residual psychosocial demands (M, sd) | 0.04 (1.49) | 0.05 (1.41) | 0.13 (1.10) | -0.42 (1.38) | 0.28 (1.39) | -0.16 (1.08) | 0.191 |
| Residual psychosocial resources (M, sd) | 0.09 (0.99) | -0.08 (0.93) | -0.06 (1.09) | 0.18 (0.90) | -0.10 (0.81) | -0.21 (0.68) | 0.117 |
|  |  |  |  |  |  |  |  |
| T1 Self-rated health (M, sd) | 2.1 (0.7) | 2.1 (0.8) | 2.0 (0.9) | 2.0 (0.8) | 2.6 (0.9) | 2.1 (0.8) | <0.001 |
| T1 Phys. limitations (% >=2) | 6.7 | 9.8 | 0.0 | 4.4 | 22.0 | 5.2 | 0.004 |
| T2 Self-rated health (M, sd) | 2.0 | 2.1 | 1.8 | 2.1 | 3.0 | 2.2 | <0.001 |
| T2 Phys. limitations (% >=2) | 8.8 | 12.9 | 0.0 | 6.7 | 40.8 | 18.2 | <0.001 |

**Table S2**. The association of pre-post exit health change with pre-exit **physical demands** by cohort, excluding disability exits (*N*=451^†^)

|  | **1990s** | | **2000s** | | **2010s** | |
| --- | --- | --- | --- | --- | --- | --- |
| ***Self-rated health (continuous)*** | | | | | | |
|  | B | 95% CI | B | 95% CI | B | 95% CI |
| **Main part of model** | | | | | | |
| Time (post- vs pre-exit) | 0.10 | -0.08; 0.27 | -0.06 | -0.20; 0.08 | -0.06 | -0.21; 0.09 |
| Physical demands (high vs low) | 0.10 | -0.13; 0.34 | 0.20 | -0.02; 0.42† | 0.17 | -0.09; 0.44 |
| Physical demands * Time | -0.14 | -0.35; 0.08 | 0.10^a^ | -0.14; 0.35 | -0.07 | -0.29; 0.15 |
| **Adjustment variables (coefficients identical for the three decades)** | | | | | | |
| Sex (female vs male) |  |  | -0.05 | -0.18; 0.09 |  |  |
| Level of education   - High vs low - Middle vs low |  |  | -0.26  -0.11 | -0.41; -0.11**  -0.24; 0.03 |  |  |
| Pre-exit working hours |  |  | -0.00 | -0.01; 0.00 |  |  |
| Exit age   - <= 58 vs 59-63 years - >= 64 vs 59-63 years |  |  | 0.04  0.03 | -0.14; 0.22  -0.12; 0.19 |  |  |
|  | | | | | | |
| ***Physical limitations: difficulty with >=2 activities*** | | | | | | |
|  | OR | 95% CI | OR | 95% CI | OR | 95% CI |
| **Main part of model** | | | | | | |
| Time (post- vs pre-exit) | 2.68 | 0.82; 8.79 | 0.73^a^ | 0.39; 1.36 | 2.42^b^ | 1.22; 4.79* |
| Physical demands (high vs low) | 0.52 | 0.04; 6.52 | 0.76 | 0.24; 2.47 | 1.84 | 0.62; 5.47 |
| Physical demands * Time | 1.61 | 0.19; 13.84 | 1.37 | 0.48; 3.98 | 0.88 | 0.32; 2.45 |
| **Adjustment variables (coefficients identical for the three decades)** | | | | | | |
| Sex (female vs male) |  |  | 0.95 | 0.51; 2.04 |  |  |
| Level of education   - High vs low - Middle vs low |  |  | 0.34  0.80 | 0.14; 0.81*  0.42; 1.54 |  |  |
| Pre-exit working hours |  |  | 0.99 | 0.96; 1.01 |  |  |
| Exit age   - <= 58 vs 59-63 years - >= 64 vs 59-63 years |  |  | 1.77  1.79 | 0.73; 4.30  0.94; 3.40† |  |  |

** p<0.001; * p<0.05; † p<0.10; B: unstandardized regression coefficient; OR: Odds Ratio; CI: Confidence Interval

^†^ 21 cases missing

^a^ Estimate differs significantly from estimate in 1990s

^b^ Estimate differs significantly from estimate in 2000s

**Table S3**. The association of pre-post exit health change with pre-exit **psychosocial demands** by cohort, excluding disability exits (*N*=451)

|  | **1990s** | | | **2000s** | | **2010s** | |
| --- | --- | --- | --- | --- | --- | --- | --- |
| ***Self-rated health (continuous)*** | | | | | | | |
|  | B | 95% CI | B | | 95% CI | B | 95% CI |
| **Main part of model** | | | | | | | |
| Time (post- vs pre-exit) | -0.04 | -0.19; 0.12 | 0.01 | | -0.15; 0.18 | -0.16^b^ | -0.31; -0.00* |
| Psychosocial demands (high vs low) | -0.18 | -0.40; 0.04 | -0.05 | | -0.27; 0.17 | -0.28^b^ | -0.54; -0.03* |
| Psychosocial demands * Time | 0.10 | -0.11; 0.31 | -0.05 | | -0.29; 0.19 | 0.16 | -0.06; 0.38 |
| **Adjustment variables (coefficients identical for the three decades)** | | | | | | | |
| Sex (female vs male) |  |  | -0.07 | | -0.20; 0.07 |  |  |
| Level of education   - High vs low - Middle vs low |  |  | -0.27  -0.19 | | -0.42; -0.12**  -0.33; -0.05* |  |  |
| Pre-exit working hours |  |  | -0.00 | | -0.01; 0.00 |  |  |
| Exit age   - <= 58 vs 59-63 years - >= 64 vs 59-63 years |  |  | 0.01  0.01 | | -0.17; 0.19  -0.15; 0.17 |  |  |
|  | | | | | | | |
| ***Physical limitations: difficulty with >=2 activities*** | | | | | | | |
|  | OR | 95% CI | OR | | 95% CI | OR | 95% CI |
| **Main part of model** | | | | | | | |
| Time (post- vs pre-exit) | 3.66 | 1.28; 10.44* | 0.94^a^ | | 0.44; 2.05 | 2.42^b^ | 1.41; 4.17** |
| Psychosocial demands (high vs low) | 1.44 | 0.35; 6.03 | 2.25 | | 0.60; 8.42 | 0.50^b^ | 0.19; 1.31 |
| Psychosocial demands * Time | 0.83 | 0.38; 1.82 | 0.83 | | 0.38; 1.82 | 0.83 | 0.38; 1.82 |
| **Adjustment variables (coefficients identical for the three decades)** | | | | | | | |
| Sex (female vs male) |  |  | 0.93 | | 0.46; 1.68 |  |  |
| Level of education   - High vs low - Middle vs low |  |  | 0.36  0.79 | | 0.15; 0.87*  0.38; 1.62 |  |  |
| Pre-exit working hours |  |  | 0.99 | | 0.96; 1.01 |  |  |
| Exit age   - <= 58 vs 59-63 years - >= 64 vs 59-63 years |  |  | 1.72  1.67 | | 0.74; 4.01  0.89; 3.14 |  |  |

** p<0.001; * p<0.05; † p<0.10

^a^ Estimate differs significantly from estimate in 1990s

^b^ Estimate differs significantly from estimate in 2000s

**Table S4**. The association of pre-post exit health change with pre-exit **psychosocial resources** by cohort, excluding disability exits (*N*=451)

|  | **1990s** | | | **2000s** | | **2010s** | |
| --- | --- | --- | --- | --- | --- | --- | --- |
| ***Self-rated health (continuous)*** | | | | | | | |
|  | B | 95% CI | B | | 95% CI | B | 95% CI |
| **Main part of model** | | | | | | | |
| Time (post- vs pre-exit) | 0.03 | -0.13; 0.20 | 0.10 | | -0.06; 0.25 | -0.07^b^ | -0.24; 0.09 |
| Psychosocial resources (high vs low) | -0.06 | -0.28; 0.17 | 0.10 | | -0.12; 0.32 | -0.16^b^ | -0.42; 0.10 |
| Psychosocial resources * Time | -0.02 | -0.24; 0.19 | -0.24 | | -0.49; -0.00* | 0.03 | -0.25; 0.19 |
| **Adjustment variables (coefficients identical for the three decades)** | | | | | | | |
| Sex (female vs male) |  |  | -0.08 | | -0.22; 0.06 |  |  |
| Level of education   - High vs low - Middle vs low |  |  | -0.26  -0.15 | | -0.41; -0.11**  -0.28; -0.01* |  |  |
| Pre-exit working hours |  |  | -0.00 | | -0.01; 0.00 |  |  |
| Exit age   - <= 58 vs 59-63 years - >= 64 vs 59-63 years |  |  | 0.02  0.01 | | -0.16; 0.20  -0.14; 0.17 |  |  |
|  | | | | | | | |
| ***Physical limitations: difficulty with >=2 activities*** | | | | | | | |
|  | OR | 95% CI | OR | | 95% CI | OR | 95% CI |
| **Main part of model** | | | | | | | |
| Time (post- vs pre-exit) | 3.32 | 0.93; 11.89† | 0.69^a^ | | 0.29; 1.67 | 2.13^b^ | 1.07; 4.24* |
| Psychosocial resources (high vs low) | 0.44 | 0.04; 5.05 | 1.08 | | 0.34; 3.41 | 0.94 | 0.32; 2.75 |
| Psychosocial resources * Time | 0.94 | 0.12; 7.59 | 1.44 | | 0.52; 4.01 | 1.14 | 0.41; 3.15 |
| **Adjustment variables (coefficients identical for the three decades)** | | | | | | | |
| Sex (female vs male) |  |  | 0.96 | | 0.47; 1.96 |  |  |
| Level of education   - High vs low - Middle vs low |  |  | 0.36  0.80 | | 0.15; 0.85*  0.43; 1.52 |  |  |
| Pre-exit working hours |  |  | 0.99 | | 0.96; 1.01 |  |  |
| Exit age   - <= 58 vs 59-63 years - >= 64 vs 59-63 years |  |  | 1.75  1.73 | | 0.72; 4.26  0.92; 3.25† |  |  |

** p<0.001; * p<0.05; † p<0.10

^a^ Estimate differs significantly from estimate in 1990s

^b^ Estimate differs significantly from estimate in 2000s

**Table S5**. The association of pre-post exit health change with pre-exit **educational level** by cohort (*N*=522)

|  | **1990s** | | | **2000s** | | **2010s** | |
| --- | --- | --- | --- | --- | --- | --- | --- |
| ***Self-rated health (continuous)*** | | | | | | | |
|  | B | 95% CI | B | | 95% CI | B | 95% CI |
| **Main part of model** | | | | | | | |
| Time (post- vs pre-exit) | 0.10 | -0.06; 0.26 | 0.16 | | -0.05; 0.38 | -0.09 | -0.35; 0.17 |
| Educational level (high vs low) | -0.02 | -0.24; 0.19 | -0.21 | | -0.44; 0.02† | -0.38^a^ | -0.36; -0.09* |
| Educational level * Time | -0.08 | -0.21; 0.52 | -0.22 | | -0.47; 0.03† | 0.08^b^ | -0.21; 0.36 |
| **Adjustment variables (coefficients identical for the three decades)** | | | | | | | |
| Sex (female vs male) |  |  | -0.15 | | -0.29; -0.01* |  |  |
| Physical demands (high vs low) |  |  | 0.19 | | 0.06; 0.31* |  |  |
| Psychosocial demands (high vs low) |  |  | -0.19 | | -0.31; -0.06*. |  |  |
| Psychosocial resources (high vs low) |  |  | -0.22 | | -0.34; -0.09** |  |  |
| Pre-exit working hours |  |  | -0.01 | | -0.01; -0.00* |  |  |
| Exit age   - <= 58 vs 59-63 years - >= 64 vs 59-63 years |  |  | 0.19  0.02 | | -0.00; 0.38†  -0.13; 0.17 |  |  |
|  | | | | | | | |
| ***Physical limitations: difficulty with >=2 activities*** | | | | | | | |
|  | OR | 95% CI | OR | | 95% CI | OR | 95% CI |
| **Main part of model** | | | | | | | |
| Time (post- vs pre-exit) | 2.49 | 0.98; 6.33† | 1.23 | | 0.70; 2.16 | 4.22^b^ | 1.51; 11.82* |
| Educational level (high vs low) | 0.44 | 0.06; 3.01 | 0.29 | | 0.10; 0.84* | 1.10^b^ | 0.36; 3.41 |
| Educational level * Time | 1.56 | 0.33; 7.46 | 0.82 | | 0.32; 2.07 | 0.40^a^ | 0.13; 1.24 |
| **Adjustment variables (coefficients identical for the three decades)** | | | | | | | |
| Sex (female vs male) |  |  | 0.75 | | 0.42; 1.36 |  |  |
| Physical demands (high vs low) |  |  | 1.14 | | 0.66; 1.99 |  |  |
| Psychosocial demands (high vs low) |  |  | 0.66 | | 0.38; 1.16 |  |  |
| Psychosocial resources (high vs low) |  |  | 0.72 | | 0.31; 1.25 |  |  |
| Pre-exit working hours |  |  | 0.98 | | 0.96; 1.00† |  |  |
| Exit age   - <= 58 vs 59-63 years - >= 64 vs 59-63 years |  |  | 1.69  1.28 | | 0.87; 3.31  0.70; 2.35 |  |  |

** p<0.001; * p<0.05; † p<0.10

^a^ Estimate differs significantly from estimate in 1990s

^b^ Estimate differs significantly from estimate in 2000s
